# Desmoid Tumor Associated With Familial Adenomatous Polyposis: Evaluation With 64-Detector CT Enterography

**DOI:** 10.5812/iranjradiol.6545

**Published:** 2012-03-25

**Authors:** Oktay Algin, Sehnaz Evrimler, Evrim Ozmen, Melike Metin, Osman Ersoy, Mustafa Karaoglanoglu

**Affiliations:** 1 Department of Radiology, Ataturk Training and Research Hospital, Ankara, Turkey; 2 Department of Gastroenterology, Ataturk Training and Research Hospital, Ankara, Turkey

**Keywords:** Fibromatosis, Aggressive, Desmoid Tumor, Familial Adenomatous Polyposis, CT Enterography, Mesentery, Fibroma

## Abstract

Desmoid tumors (DTs) are benign tumors which are not seen very often, and most of the radiologists and clinicians do not know the characteristics of them very well. Correct and early diagnosis of DTs is important for decreasing mortality and morbidity. Computed tomography enterography (CTE) is a new modality for small bowel imaging which combines the improved spatial and temporal resolution of multidetector computed tomography (CT) with large volumes of ingested enteric contrast material to permit evaluation of the small bowel wall and lumen and also the entire abdomen. We report a familial adenomatous polyposis (FAP) patient with localized mesentery and abdominal wall DTs. We showed the exact location of the DTs and their relation with the small bowel by CTE. In conclusion, CTE is a useful technique for DT localization, the degree of extension and invasion to local structures, presence of partial and complete small bowel obstruction, and the relationship of the tumors with vasculature and whether ischemia has occurred as a result or not.

## 1. Introduction

Desmoid tumors (DTs) are uncommon benign tumors occurring as a result of excessive proliferation of connective tissue which tend to be locally more invasive than metastasis [1]. Thirty percent of patients with DTs have the diagnosis of familial adenomatous polyposis (FAP), but DTs can be seen sporadically as well [1]. DTs generally occur after prior abdominal surgery (especially total colectomy) in FAP patients [2]. Mesenteric DTs may lead to small bowel obstruction, ischemia, fistula formation, hydronephrosis and bowel perforation [1]-[4]. Therefore, accurate and early diagnosis and treatment are important for decreasing mortality and morbidity [5]. Besides, determination of the relation between DTs and the surrounding tissues is important for surgical planning [5].

In routine clinical practice, conventional abdominal computed tomography (CT) or magnetic resonance imaging (MRI) is used in the diagnosis of mesenteric DT [2]. Abdominal roentgenograms, enteroscopy, capsule endoscopy and/or conventional enteroclysis could give indirect information for DT diagnosis, but they are frequently inadequate and have several limitations as well.

CT enterography (CTE) is a new modality for small bowel imaging which combines the improved spatial and temporal resolution of multidetector CT (MDCT) with large volumes of ingested enteric contrast material to permit visualization of the small bowel wall and lumen and also the entire abdomen [6][7]. Different from routine abdominal CT and MRI, CTE can be useful for the evaluation of mesenteric DTs and the relationship of the tumor with small bowel.

We report a FAP patient with mesenteric and abdominal wall DTs. We showed their exact location and relation with the small bowel by CTE. As far as we know, there has not been a report of a patient with DT who was diagnosed with CTE in the literature. Our aim is to discuss the efficacy of CTE in the detection and evaluation of DTs referring to the literature. We think that this report may be useful for early and correct diagnosis of similar cases.

## 2. Case Presentation

The patient, who was diagnosed as FAP and had total colectomy before, admitted to the gastroenterology clinic of our hospital with abdominal pain and anemia. In order to understand the etiology of anemia, upper gastrointestinal endoscopy was performed and neither the origin of the bleeding nor a cause such as malignancy or any other etiology was detected. Therefore, we decided to use CTE in this case to see the possible existence of any intra- or extraintestinal pathologic condition.

Before CTE, 1250 mL of oral contrast material solution–775 mL of water, 250 mL of lactulose (667 mg/mL, Osmolac, Biofarma, Turkey) and 225 mL of dilute barium sulfate suspension especially designed for abdominal MDCT examinations (E-Z-Cat, Opakim, Turkey)–was ingested over 45 min at a steady rate. After administration of the oral contrast agent, CTE was performed with 64-detector MDCT machine (Aquilion 64, Toshiba, Tokyo, Japan). After intravenous administration of 100 mL of Iopromide (Ultravist, Schering, Germany) at a flow rate of 3 mL/s, abdominal contrast-enhanced MDCT images with 1 mm slice thickness were obtained in portal phase. After CTE examination, sagittal and coronal reformatted images (slice thickness: 1 mm) were obtained which are routinely available on workstation inside the MDCT machine.

**Figure 1:**
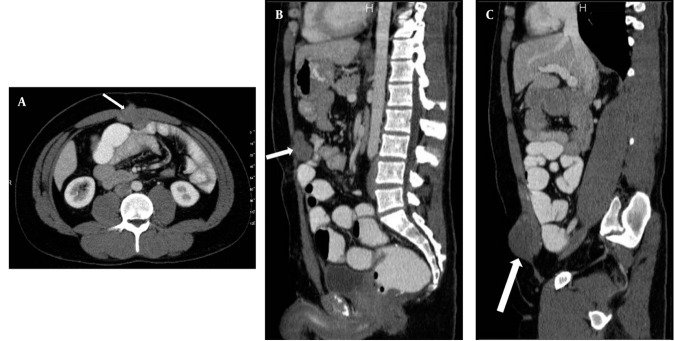
A, Axial cut obtained at the superior level of the umbilicus; B, Reformatted midline sagittal; and C, Right parasagittal CTE images of the patient show anterior abdominal wall desmoid tumors (DTs) (arrows). The relationship between DTs and small bowel loops is clearly seen on the MDCT images.

**Figure 2:**
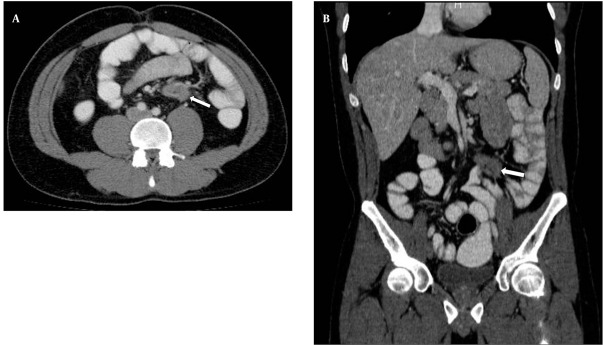
A, Axial; and B, Reformatted coronal CTE images of the patient. Morphology of mesenteric desmoid tumor (arrows) and the relationship between the tumor and the small bowel-mesenteric vasculature is seen on MDCT images. In addition, small bowel ischemia, invasion, obstruction or any other complication of desmoid tumors was not detected.

In CTE examination, there was no sign of manifested small bowel obstruction and intraluminal pathology. Intraabdominal solid organs were normal. A 39 × 42 × 36 mm sized contrast material enhancing solid mass was seen between the rectus muscles at the superior region of the umbilicus, invading to the subcutaneous fatty tissue anteriorly and limited by the omentum posteriorly. This mass had a well-defined margin making indentation without invasion to the small bowel loops (Figure 1). Another similar solid mass 60 × 48 × 35 mm in size was seen in the right inguinal region, anterolateral to the right rectus muscle, similarly indentated small bowel loops and limited by the omentum. Adjacent to the posterior segment of the eleventh left rib, a similar solid subcutaneous lesion (approximately 13 mm in diameter) was observed. In addition, another 38 × 33 × 29 mm sized, hyper-dense mass with lobulated contour was seen at the small bowel mesentery, being close to the ileal branches of the superior mesenteric artery (SMA). It is observed that, this mass surrounded the SMA branches, but did not cause a significant obstruction in the arterial flow (Figure 2). Small bowel segments close to the defined mass were detected as normal. According to CTE results, this mass was interpreted as a mesenteric DT.

As a result of these findings, gray scale and Doppler ultrasound were applied. On gray scale US, the masses showed well-circumscribed homogeneous hypoechoic appearance (character of fibrosis). In the anterior abdominal wall, two lesions, which were close to the peritoneum and greater omentum, were localized. On Doppler US imaging of these lesions, no sign of significant vascularization was seen. Mesenteric DT seen in CTE could not be demonstrated by US. As a result of US and CTE findings, all these lesions are considered as DTs. Thus, medical therapy and follow-up was recommended.

## 3. Discussion

DTs, also called abdominal fibromatosis are benign tumors which are not seen very often, and most of the radiologists and clinicians do not know the characteristics of them very well [1][2]. The etiology of DT is genetic predisposition (in patients with FAP or Gardner syndrome), trauma, prior abdominal surgery and hormonal factors (endogenous level of estrogen, pregnancy) [4]. DTs are generally seen between 20 and 40 years of age as in our case and generally occur after colectomy in patients with FAP or Gardner syndrome [1][2]. DTs are locally aggressive tumors which do not metastasize, but recurrence ratios are high especially in patients with FAP [2]. The small bowel mesentery, musculoaponeurotic structures of the anterior or posterior abdominal wall, especially the rectus and oblique muscles and their fascial coverings are the most common sites of origin for DTs [1][2][4].

DTs are often asymptomatic, but can represent as abdominal pain, palpable abdominal mass, nausea, anemia, vomiting, diarrhea or fever [1][2][8]. DT complications are partial or complete obstruction of the small bowel or ureter as a result of intestinal or ureteral compression, intestinal perforation with or without peritonitis, abscess and fistula formation [1][2][4][8]. Small bowel ischemia, mucosal ulcerations and/or gastrointestinal bleeding occur as a result of increased compression or invasion of mesenteric vessels [1][2][4][8].

The treatment options for DTs include medical therapy (e.g. non-steroidal anti-inflammatory drugs, antiestrogens), cytotoxic chemotherapy, radiation therapy or surgery [1][2]. Due to a higher recurrence risk and more difficult disease control, non-invasive treatment methods should be planned in the first place for FAP patients with DT different from sporadic cases [1]. Surgical treatment option should be kept only for patients in whom complication has occurred (e.g. small bowel obstruction or perforation) [2]-[5].

The radiologic characteristics of DTs could be changed according to their fibroblastic proliferation, collagen component, vascularity and fibrosis [1][2][8]. On US, DTs have variable echogenicity and homogeneity with or without smooth, well-defined margins [8]. US which is not the first option for mesenteric DT evaluation can be useful for detecting abdominal wall localized DTs. Thus, CT and MRI should be preferred for DT assessment in the first line [1].

On contrast-enhanced abdominal CT scans, most DTs appear as homogeneous masses with well- or ill-defined margins that may have iso- or hyperdense appearance relative to muscle planes [1][2]. Some cases of heterogeneous masses with infiltrative outer margins have been seen [2]. DTs may enhance after injection of IV contrast material [1]. On MRI, DTs appear as masses with low signal intensity relative to muscle planes on T1 weighted (W) sequences [2]. DTs could show various signal characteristics on T2W sequences due to their hysto-pathological features [8]. In summary, radiological characteristics of these tumors are directly related to their underlying histopathologic features and vascularity [1]. Therefore, there is no significant imaging criterion for DTs. Lymphomas, mesenteric sarcomas and gastrointestinal stromal tumors should be considered in the differential diagnosis of DTs from other mesenteric and abdominal solid lesions. Nonetheless, first of all, DTs should be considered for mesenteric and anterior abdominal wall masses in patients with FAP or Gardner syndrome and cases with surgery or trauma history [2].

CTE is a relatively new technique which is used generally in patients with Crohn’s disease and other small bowel pathologies [9][10]. The difference between CTE and conventional and CT enteroclysis is that; there is no need for nasojejunal intubation and as a result, there is better patient comfort and less radiation exposure [9][11]. Colectomy is the preferred operation for patients with polyposis syndrome as performed in our patient. Providing luminal distension using routine oral contrast agents is almost impossible in such patients due to the short intestinal transit time. The oral contrast agent which was used for CTE examination was developed for small bowel distension. Thus, the optimal luminal distension could be achieved and the relation of DTs with the small bowel and mesenteric structures could be clearly demonstrated.

The advantages of CTE with MDCT are scanning the whole abdomen with thin slices in one breath time, taking multiplanar reformatted images and showing the small bowel and the surrounding tissues directly [12][13]. The disadvantage of CTE and the difference from MR enterography (MRE) is the radiation exposure [9]. Thus, MRE can be a new follow up modality in the evaluation of young patients [9][12]. However, MRE has its own limitations and disadvantages, as it cannot be applied for claustrophobic or uncooperative patients and is highly sensitive to movement and post-operative metallic materials [9].

As a result of the developments in CT technology, there has been significant improvement in reformatted image quality. We could evaluate the extension of mesenteric DTs, surrounding the small bowel and the vessels with 64 detector MDCT which could not be detected by US or endoscopy. Besides, we could easily determine the relationship between anterior abdominal wall localized DTs and the small bowel or the intraperitoneal space with CTE. New and comprehensive studies are needed to determine the role of CTE in demonstrating the relation of DTs with the small bowel and mesenteric structures. The effect on the prognosis and also the role of the morphological appearance of DTs in enterographic images on the surgical plan should be assessed in detail in those further studies.

According to the results of our case, CTE is a useful technique for the evaluation of DT location, degree of extension and invasion to local structures, or the presence of partial/complete small bowel obstruction and the relationship of the tumors with vasculature and whether ischemia has occurred as a result or not. CTE is good also for examining extra-intestinal complications such as fistulas, urinary tract obstruction and abscess formation.
